# In this issue: Volume 115, Issue 6, May 2024

**DOI:** 10.1111/cas.16231

**Published:** 2024-06-03

## Impacts of tissue context on extracellular vesicles‐mediated cancer–host cell communications

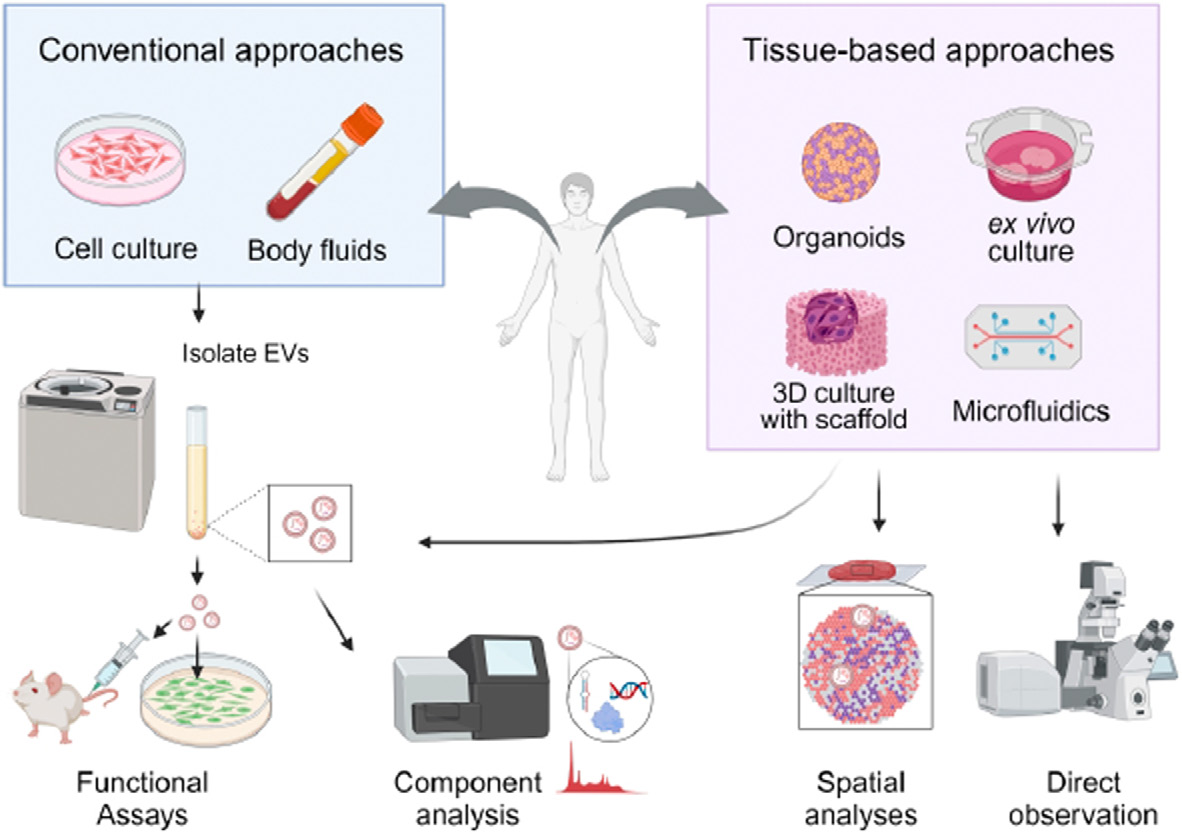

Tumor tissues are complex environments that contain cancer cells, normal cells, and a network of supporting molecules, called the extracellular matrix (ECM). Within this mix, cancer cells release tiny packages called extracellular vesicles (EVs) that can transform nearby normal cells into cells that support tumor growth. Understanding the interactions between these EVs and surrounding cells is a crucial aspect of cancer research. Despite their importance, most research on EVs focuses on studying them outside of tumor tissues, which doesn't fully capture how EVs behave within actual tumor tissues.

Now, researchers Nao Nishida‐Aoki and Takahiro Ochiya reviewed how tumor tissue environment likely alters EV behavior by changing their properties, creating physical barriers, and influencing target cell interactions. Advanced techniques are needed to investigate EV movements and analyze their effects at a cellular level within the tissue. Although detecting EV dynamics is tough due to their small size, the authors suggest introducing advanced imaging methods, like fluorescent labeling, to track EV transfer in living animals like mice and zebrafish or tissue models prepared in the lab. Moreover, new methods like label‐free imaging and reporter systems will facilitate the study of EV‐mediated interactions within tumors. These approaches provide valuable insights into EV behavior in tissue environments.

This review article aims to highlight the mechanisms by which EVs operate within tissue environments, offering broader insights into cellular interactions beyond cancer. EVs are fundamental to how cells communicate, not only in cancer but also in normal physiological processes and even in interactions between different organisms. Studying basic mechanisms of EV transfer in tumors can shed light on how similar processes might occur in healthy tissues or even in interactions between different species, like those between microbes and the human gut.

Ultimately, this knowledge could pave the way for innovative treatments that disrupt these harmful communications, which could have wide‐ranging implications for both cancer treatment and our understanding of broader biological processes.

Link to the original journal article: https://onlinelibrary.wiley.com/doi/10.1111/cas.16161

## *Rev1* overexpression accelerates *N*‐methyl‐*N*‐nitrosourea (MNU)‐induced thymic lymphoma by increasing mutagenesis

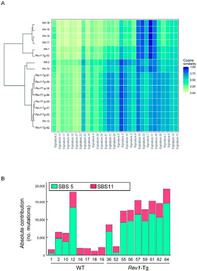

DNA replication is an essential process for biological inheritance, ensuring the accurate copying of DNA to maintain genomic stability. However, this process is prone to many accidental errors or lesions. Such lesions usually stall DNA replication and can sometimes have deleterious effects. To avoid this, cells have an in‐built damage tolerance pathway, known as translesion synthesis, which allows them to bypass DNA damage and overcome this hurdle, thereby promoting cell survival.

Rev1, a polymerase, is a key component of translesion synthesis. Previous studies investigating the functions of this polymerase have demonstrated that Rev1 promotes cell survival after exposure to DNA‐damaging agents through its deoxycytidyl transferase activity. Additionally, the C‐terminal domain of Rev1 interacts with other polymerases, acting as a molecular scaffold during translesion synthesis. These studies highlight Rev1's roles in maintaining the integrity of the DNA in the presence of lesions.

However, the role of Rev1 in mutation and cancer progression remains unclear. To address this, Sasatani et al. aimed to uncover the role of *Rev1* overexpression in mouse models. In their previous research, the scientists generated *Rev1* overexpressing mice (*Rev1*‐Tg) and observed that these mice showcased higher incidences of developing intestinal adenoma or thymic lymphoma (TL) after treatment with N‐methyl‐N‐nitrosourea (MNU), a carcinogenic agent.

In this study, they utilized various approaches, like whole‐genomic sequencing, to gain insights into *Rev1* overexpression in chemically induced tumorigenesis.

The whole‐genomic sequencing approach revealed that the mutations occurred more frequently in the *Rev1*‐Tg mice than in the wild type. Further investigations showed that the mutation pattern depended on the number of mutations per TL rather than on the genotype of the mice. They also found that the mouse TL mutations were similar to those associated with aging and alkylating agents.

In conclusion, this study shows that *Rev1* overexpression speeds up mutagenesis and increases TL incidence by decreasing the time from disease emergence to clinical emergence. This may be associated with more frequent DNA damage‐induced genetic instability. Future studies in this area can help develop a better understanding of the mechanisms by which Rev1 participates in the DNA damage response and its function in cancer progression in specific organs.

https://onlinelibrary.wiley.com/doi/10.1111/CAS.16159

## IGSF3 is a homophilic cell adhesion molecule that drives lung metastasis of melanoma by promoting adhesion to vascular endothelium

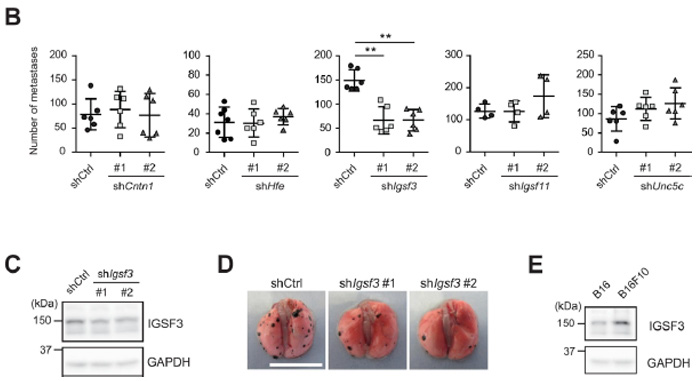

Metastasis, the spread of cancer cells from one part of the body to another, poses a significant challenge in treating solid tumors like melanoma. Interactions between tumor cells and the host microenvironment, particularly stromal cells, are pivotal in driving metastasis. This process involves several steps, including invasion, survival in circulation, migration to distant organs, and growth at secondary sites. Understanding these mechanisms is crucial for developing effective treatment strategies.

The study focuses on exploring the specific role of the immunoglobulin superfamily (IgSF), a diverse group of molecules involved in cancer progression, in lung metastasis. To this end, researchers aimed to identify IgSF molecules involved in promoting metastasis using experimental lung metastasis models. First, they compared the gene expression of highly metastatic B16F10 melanoma cells to their less aggressive counterparts, the parental B16 cells. Their analysis revealed that a particular molecule called IGSF3 was significantly more expressed in the highly metastatic B16F10 cells.

Furthermore, they conducted experiments both in in vitro as well as in mouse models of lung metastasis to investigate the function of IGSF3. They specifically looked at how IGSF3 plays a role in the attachment of melanoma cells to vascular endothelial cells (vECs), cells lining the inner surface of blood vessels, and their subsequent movement into the lungs.

This experiment revealed that IGSF3 plays a critical role in promoting the attachment of melanoma cells to vECs and facilitating their movement into the lungs, a key step in the metastatic process. These findings highlight the potential of targeting IGSF3, to hinder melanoma metastasis. Additionally, the analysis of human melanoma datasets identified a correlation between elevated IGSF3 levels and poorer patient prognosis, indicating its potential as a prognostic marker.

In conclusion, this study sheds light on the critical role of IGSF3 in driving melanoma metastasis, highlighting its potential as both a therapeutic target and a prognostic marker. Further research might reveal the mechanism of IGSF3‐mediated interactions to develop improved treatments and outcomes for melanoma patients. Moreover, the study lays the groundwork for exploring similar mechanisms in other cancers, offering promising avenues for future research.

https://onlinelibrary.wiley.com/doi/10.1111/CAS.16166

